# Regulation and functions of the NLRP3 inflammasome in RNA virus infection

**DOI:** 10.3389/fcimb.2023.1309128

**Published:** 2024-01-05

**Authors:** Zhaoyang Yue, Xuelong Zhang, Yu Gu, Ying Liu, Lin-Miaoshen Lan, Yilin Liu, Yongkui Li, Ge Yang, Pin Wan, Xin Chen

**Affiliations:** ^1^ Institute of Medical Microbiology, College of Life Science and Technology, Jinan University, Guangzhou, China; ^2^ Key Laboratory of Viral Pathogenesis & Infection Prevention and Control (Jinan University), Ministry of Education, Guangzhou, China; ^3^ Foshan Institute of Medical Microbiology, Foshan, China; ^4^ Wuhan Institute of Biomedical Sciences, School of Medicine, Jianghan University, Wuhan, China

**Keywords:** inflammation, RNA virus, NLRP3 inflammasome, pyroptosis, therapeutic strategy

## Abstract

Virus infection is one of the greatest threats to human life and health. In response to viral infection, the host’s innate immune system triggers an antiviral immune response mostly mediated by inflammatory processes. Among the many pathways involved, the nucleotide-binding oligomerization domain (NOD)-like receptor protein 3 (NLRP3) inflammasome has received wide attention in the context of viral infection. The NLRP3 inflammasome is an intracellular sensor composed of three components, including the innate immune receptor NLRP3, adaptor apoptosis-associated speck-like protein containing CARD (ASC), and the cysteine protease caspase-1. After being assembled, the NLRP3 inflammasome can trigger caspase-1 to induce gasdermin D (GSDMD)-dependent pyroptosis, promoting the maturation and secretion of proinflammatory cytokines such as interleukin-1 (IL-1β) and interleukin-18 (IL-18). Recent studies have revealed that a variety of viruses activate or inhibit the NLRP3 inflammasome via viral particles, proteins, and nucleic acids. In this review, we present a variety of regulatory mechanisms and functions of the NLRP3 inflammasome upon RNA viral infection and demonstrate multiple therapeutic strategies that target the NLRP3 inflammasome for anti-inflammatory effects in viral infection.

## 1 Introduction

The innate immune response is a natural immune defense mechanism that was gradually formed during evolution. It is considered the first line of defense against pathogen invasion. Innate immune responses are vital for eliminating external pathogens as well as for generating a powerful adaptive immune response (Medzhitov and Janeway, 1997; Medzhitov, 2007; Koyama et al., 2008).

Pattern recognition receptors (PRRs) are highly conserved sensors that quickly detect viral infection and launch antiviral immune responses (Tan et al., 2018). Inflammasome is an important member of PRR. At present, there are four main types of inflammasome, mainly including: pyrin domain containing 1 (NLRP1), absent in melanoma 2 (AIM2), pyrin domain containing 3 (NLRP3) and caspase activation recruitment domain containing 4 (NLRC4) (Broz and Dixit, 2016; Guo et al., 2018). Many different types of PAMPs, DAMPs, and environmental irritants might trigger their activation (Schroder and Tschopp, 2010). The NLRP1 can be activated by lethal toxin (LeTx) (Boyden and Dietrich, 2006); AIM2 can be activated by dsRNA (Bürckstümmer et al., 2009; Hornung et al., 2009); activation of the NLRP3 inflammasome is triggered by a wide range of stimuli, including microbial infections, cellular stress, tissue damage, and some metabolic disturbances (Kelley et al., 2019; Wang and Hauenstein, 2020). NLRC4 can be activated by Salmonella typhimurium, Legionella pneumophila (Legionella), etc. (Mariathasan et al., 2004; Schroder and Tschopp, 2010).

The nucleotide-binding oligomerization domain (NOD)-like receptor protein 3 (NLRP3) inflammasome plays a vital role in innate immune responses and has garnered considerable attention in the context of virus infection (Wang et al., 2014; He et al., 2016). The NLRP3 inflammasome is composed of three components: the innate immune receptor NLRP3, the effector cysteine protease caspase-1, and the adaptor apoptosis-associated speck-like protein containing a C-terminal caspase recruitment domain (CARD) (ASC). To initiate inflammatory responses and activate the inflammasome, NLRP3 identifies pathogen-associated molecular patterns (PAMPs) and danger-associated molecular patterns (DAMPs) (Jin and Flavell, 2010; Kelley et al., 2019). It has three different domains: a central nucleotide-binding and oligomerization domain (NOD), an amino-terminal pyrin (PYD) domain, and a carboxy-terminal leucine-rich repeat (LRR) domain. These three domains play vital roles in NLRP3 activation. The PYD domain recruits the pyrin domain of ASC to initiate the assembly of the inflammasome; on the other hand, the NOD domain, which exhibits ATPase activity, is vital for NLRP3 oligomerization after activation; the LRR domain is the region that recognizes ligands (Schroder and Tschopp, 2010). The activation of the NLRP3 inflammasome results in the maturation of proinflammatory cytokines and secretion of active interleukin (IL)-1β and IL-18; these cytokines subsequently trigger downstream immune responses and inflammation (Sagulenko et al., 2013).

**Figure 1:**
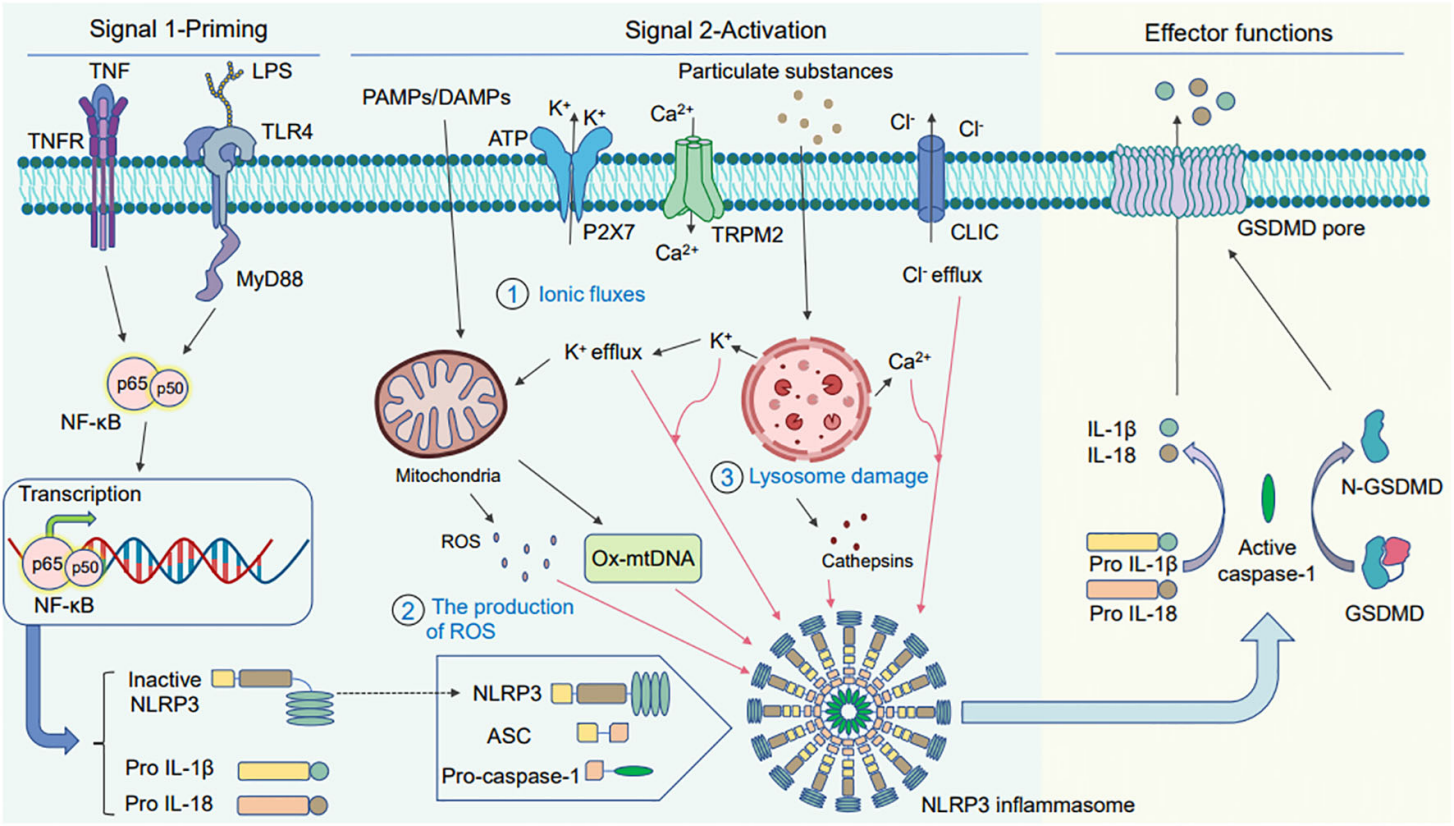
Schematic diagram of the two signaling pathways warranted for activating the NLRP3 inflammasome. Two signals are needed for activating the NLRP3 inflammasome. The first is the priming signal: tumor necrosis factor (TNF) and lipopolysaccharide act on the cell surface receptors TNFR and Toll-like receptor (TLR) 4, which activate the nuclear factor- kappaB (NF-κB) pathway and induce gene expression to produce large amounts of pro-IL-1β, pro-IL-18, and NLRP3. The second is the activation signal: pathogen-associated molecular patterns (PAMPs) and danger-associated molecular patterns (DAMPs) induce the assembly and activation of the NLRP3 inflammasome. There are three main DAMP models: (1) ionic flux model, (2) reactive oxygen species (ROS) production, and (3) lysosomal damage. The activated NLRP3 inflammasome activates caspase-1, pro-IL-1β, and pro-IL-18 to produce active IL-1β and IL-18 under the action of active caspase-1, finally releasing them outside the cell. In addition, activated caspase-1 can also cleave GSDMD to N-GSDMD and liberate them to insert into membrane and form the pyroptotic pores. The mature IL-1β and IL-18 release out of the cell together with cell content through GSDMD-pores to induce pyroptosis.

NLRP3 inflammasome activation occurs via two steps: signal 1 priming and signal 2 activation (**Figure 1**) (Sutterwala et al., 2014; He et al., 2016; Abad and Danthi, 2020). In the first step, NLRP3 and active IL-1β expression is upregulated via the Toll-like receptor (TLR) and nuclear factor-kappaB (NF-κB) signaling pathways. This is regulated at the transcriptional and translational levels (Bauernfeind et al., 2009). Under the action of microbial components or endogenous cytokines such as tumor necrosis factor (TNF) and lipopolysaccharide, the transcription factor NF-κB is activated via receptors such as TLR4 or TNF receptor superfamily (TNFR), resulting in the transcription of NLRP3, pro-caspase-1, pro-IL-1β, and pro-IL-18 (Sutterwala et al., 2014). In the second step, the NLRP3 inflammasome is activated via PAMPs and DAMPs, where NLRP3, caspase-1, and ASC combine to form the NLRP3 inflammasome, subsequently activating caspase-1 to drive the maturation and secretion of IL-1β. This encompasses the regulation of NLRP3 inflammasome activation at the post-translational level (Bauernfeind et al., 2009; Cai et al., 2014). Tissue injury, metabolic imbalance, or different stress signals such as ATP, particulate matter, bacteria, and viruses can activate the NLRP3 inflammasome. Furthermore, the stimulation can be activated via three modes: potassium efflux (K^+^ efflux), lysosomal destabilization, and reactive oxygen species (ROS) production (Pétrilli et al., 2007; Dostert et al., 2008; Halle et al., 2008; Tschopp and Schroder, 2010; Lamkanfi and Dixit, 2014; Swanson et al., 2019). Once activated, NLRP3 can be further linked to the ASC via its PYD domain. ASC binds to pro-caspase-1 via its CARD domain to form a complete structure; thereafter, it forms apoptosis-related spot-like proteins to form inflammasome complexes. Active caspase-1 is produced by autocatalytic cleavage, and subsequently activated caspase-1 catalyzes the transcription of IL-1β precursor proteins to produce active IL-1β (Fernandes-Alnemri et al., 2007; Franchi et al., 2009; Latz, 2010). This suggests that activating the NLRP3 inflammasome is a complicated process involving multiple cellular events. Consequently, further elucidations are needed to understand the regulatory processes of NLRP3 inflammasome activation.

When the NLRP3 inflammasome is activated by virus infection, it disrupts the replication niche within the pathogen cell, thereby promoting the release of proinflammatory factors; this, in turn, leads to the activation of a highly inflammatory form of cell death, namely pyroptosis; subsequently, it plays a barrier role in innate immunity (Miao et al., 2010; Shi et al., 2017). Pyroptosis is an innate immune effector mechanism in the host antiviral defense system and a type of lytic cell death that further triggers the inflammatory cascade and activates immune surveillance systems to promote virus clearance (Vande Walle and Lamkanfi, 2016; de Vasconcelos et al., 2019; Imre, 2020). Activated caspase-1 cleaves gasdermin D (GSDMD) to generate independent N- and C-terminal fragments. The N-terminal fragment of GSDMD induces osmotic pressure imbalance and membrane rupture by forming plasma membrane pores, thereby facilitating cell leakage and dissolution and extracellular active IL-1β secretion (Fink and Cookson, 2006; Sborgi et al., 2016; Evavold et al., 2018; Huang et al., 2021).

RNA viruses and emerging human pathogens are public health concerns. Various RNA viruses have developed various strategies to evade innate immune responses; they can induce the production of inflammatory factors that can lead to fatal diseases (Nelemans and Kikkert, 2019). However, whether the virus can activate or inhibit the NLRP3 inflammasome remains controversial. Therefore, we elucidate and discuss the regulatory relationship between the NLRP3 inflammasome and RNA virus infection. Herein, we classified RNA viruses according to positive-sense single-stranded, negative-sense single-stranded and double-stranded RNA viruses. The various mechanisms by which RNA viruses regulate the activation or inhibition of the NLRP3 inflammasome were summarized (**Table 1**). We briefly discuss the clinical significance of NLRP3 inflammasome activation in viral disease. We also discuss several therapeutic strategies targeting the NLRP3 inflammasome for anti-inflammatory effects in virus infection. The hope is to provide valuable experience for the clinical treatment of NLRP3-related diseases caused by RNA viruses.

**Table 1:** Regulatory mechanism of the NLRP3 inflammasome in RNA virus infection.

Viruses	Regulatory mechanism	Reference
Severe acute respiratory syndrome coronavirus (SARS-CoV)	1. Signal 1-Priming: ORF3a, E 2. Signal 2-Activation: E, ORF3a, ORF8b	(DeDiego et al., 2014; Nieto-Torres et al., 2015; Chen et al., 2019; Shi et al., 2019; Siu et al., 2019).
Severe acute respiratory syndrome coronavirus-2 (SARS-CoV-2)	1. Signal 1-Priming: S, ORF3a 2. Signal 2-Activation: ORF3a, N, C3a, MAC	(Asgari et al., 2013; Sluyter, 2017; Magro et al., 2020; Ratajczak and Kucia, 2020; Olajide et al., 2021; Pan et al., 2021; Zhao et al., 2021; Xu et al., 2022; Triantafilou et al., 2013a)
Dengue virus (DENV)	1. Signal 1-Priming: DENV viral RNA, E(EDIII) 2. Signal 2-Activation: E(EDIII), NS2A, NS2B, M,	(Khan et al., 2019; Pan et al., 2019a; Shrivastava et al., 2020a; Shrivastava et al., 2020b)
Zika virus (ZIKV) Enterovirus 71 (EV71)	1. Signal 2-Activation: NS5, 2. Inhibition mechanism: NS3	(He et al., 2018; Wang et al., 2018; Gim et al., 2019)
Enterovirus 71 (EV71)	1. Signal 1-Priming: VIM-ERK-NF-κB 2. Signal 2-Activation: 3D, TXNIP 3. Inhibition mechanism: 2A, 3C	(Wang et al., 2015; Wang et al., 2017; Xiao et al., 2019; You et al., 2020; Qayyum et al., 2021; Gong et al., 2022; Hu et al., 2023)
Human Immunodeficiency Virus (HIV)	1. Signal 1-Priming: HIV-1, Vpr, Tat 2. Signal 2-Activation: Tat, HIV-1 RNA, E	(Demarchi et al., 1996; Benelli et al., 2000; Séror et al., 2011; Lee et al., 2012; Hernandez et al., 2014; Chivero et al., 2017; Paoletti et al., 2019; He et al., 2020; Li et al., 2020; Stunnenberg et al., 2021)
Influenza virus (FLU)	1. Signal 1-Priming: NP, RNA 2. Signal 2-Activation: M2, PB1-F2 protein, RNA 3. Inhibition mechanism: NS1	(Allen et al., 2009; Ichinohe et al., 2009; Ichinohe et al., 2010; Pang and Iwasaki, 2011; McAuley et al., 2013; Yoshizumi et al., 2014; Chung et al., 2015; Moriyama et al., 2016; Kuriakose and Kanneganti, 2017; Moriyama et al., 2019; Wang et al., 2021; Kim et al., 2022)
Respiratory syncytial virus (RSV)	1. Signal 2-Activation : SH, ORMDL3	(Triantafilou et al., 2013b; Cantero-Recasens et al., 2010)

## 2 Regulatory mechanisms and functions of the NLRP3 inflammasome during RNA virus infection

### 2.1 Positive-sense single-stranded RNA virus

#### 2.1.1 Human coronaviruses

Coronaviruses belong to the family *Coronaviridae* and comprise a positive-sense single-stranded RNA that is 27–32 kb long (Van Der Hoek et al., 2004; Lu et al., 2020). Both severe acute respiratory syndrome coronavirus (SARS-CoV) and novel severe acute respiratory syndrome coronavirus-2 (SARS-CoV-2) are types of human coronaviruses (Hasöksüz et al., 2020). The genomes of SARS-CoV and SARS-CoV-2 encode four structural proteins: spike (S), envelope (E), membrane (M), and nucleocapsid (N) proteins. The RNA genome is encapsulated by the N proteins. The M and E proteins ensure that the N proteins can be incorporated into the virus particles during assembly, whereas the S proteins provide specificity for cellular entry receptors (Alanagreh et al., 2020; Kim et al., 2020; Liu et al., 2020a). After binding to specific receptors such as angiotensin-converting enzyme 2, coronaviruses can either directly fuse with the cell surface or be engulfed by the endosomes, thereby entering the cell. Then, the viral N protein enters the cytoplasm, releasing the RNA genome of the virus into the cell. It synthesizes new virus RNA and proteins and assembles them into virus particles for release (Fehr and Perlman, 2015; Hartenian et al., 2020; Prydz and Saraste, 2022).

##### 2.1.1.1 SARS-CoV virus infection and NLRP3 inflammasome

SARS-CoV-encoded proteins can activate the NLRP3 inflammasome via the NF-κB pathway. For example, ORF3a and E proteins play vital roles in activating the NLRP3 inflammasome. ORF3a and E proteins of SARS-CoV can activate NF-κB, resulting in the transcription of pro-IL-1β (DeDiego et al., 2014; Siu et al., 2019). Furthermore, ORF3a promotes the ubiquitination of p105 and ASC via TNFR-associated factors, promoting the activation of NF-κB and the NLRP3 inflammasome (Siu et al., 2019).

ORF3a and E proteins of SARS-CoV play roles in the second activation step. ORF3a can lead to lysosomal dysfunction in the host cells and initiate caspase-1 activation either directly or via increased K^+^ efflux. The ion channel function of ORF3a is vital for activating the NLRP3 inflammasome, mitochondrial ROS production, and K^+^ efflux during active IL-1β secretion (Chen et al., 2019). On the other hand, E proteins can alter the permeability of Ca^2+^ in the plasma membrane to activate the inflammasome. To be specific, Ca^2+^ leakage via the E protein ion channels and increased cytoplasmic Ca^2+^ levels may activate the NLRP3 inflammasome in the endoplasmic reticulum–Golgi intermediate compartment/Golgi membranes (Nieto-Torres et al., 2015). In addition, the activation of the inflammasome involves other proteins. In macrophages, ORF8b directly interacts with the LRR domain of NLRP3 and co-localizes with NLRP3 and ASC in the cytosol for activation (Shi et al., 2019).

##### 2.1.1.2 SARS-CoV-2 virus infection and NLRP3 inflammasome

**Figure 2:**
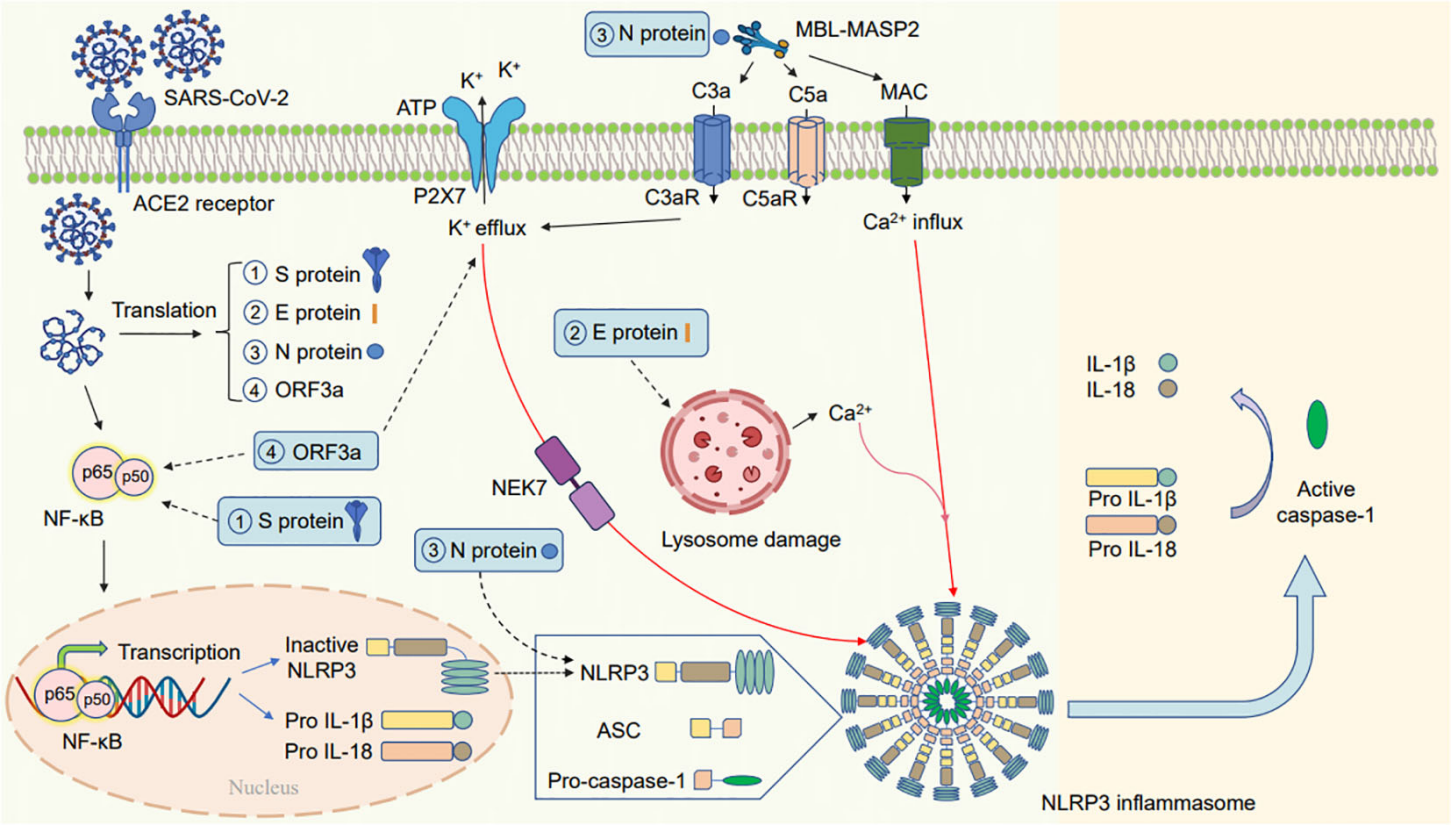
Schematic diagram of the mechanism by which SARS-CoV-2 activates the NLRP3 inflammasome. SARS-CoV-2 infection may activate the NLRP3 inflammasome in the following ways: (1) one of the subunits of the spike glycoprotein S1 can release proinflammatory cytokines via mechanisms involving the activation of the NF-κB pathway. (2) The E protein of SARS-CoV-2 can cause lysosomal damage to release a large amount of Ca^2+^, thereby activating the NLRP3 inflammasome. (3) The N viral protein interact with NLRP3 to promote inflammasome assembly; the complement cascade induced by the N protein–MBL-MASP2 axis may lead to the activation of the NLRP3 inflammasome via the different functions of C3a, C5a, and membrane attack complex (MAC). (4) ORF3a primes the inflammasome via NF-κB-mediated transcriptional activation of pro-IL-1β. Furthermore, ORF3a activates the NLRP3 inflammasome via K^+^ efflux and NEK7. The activated NLRP3 inflammasome can activate transcription of pro-IL-1β and pro-IL-18 and produce mature IL-1β and IL-18.

Some patients with severe COVID-19 experience a cytokine storm and exhibit inflammasome activation; this increases the amount of active IL-1β and IL-18 in the lungs, cerebrospinal fluid, and serum (Conti et al., 2020; Guasp et al., 2022). The mechanism of activation of the NLRP3 inflammasome by SARS-CoV-2 infection is depicted in **Figure 2**.

When infected with SARS-CoV-2, the NF-κB pathway is activated, leading to the increased expression and production of NLRP3 and active IL-1β. For instance, the SARS-CoV-2 spike glycoprotein S1 activates NF-κB, p38 MAPK, and the NLRP3 inflammasome, leading to the release of proinflammatory cytokines in peripheral blood mononuclear cells (Olajide et al., 2021). Furthermore, ORF3a of SARS-CoV-2 can activate the NLRP3 inflammasome by triggering pro-IL-1β transcription via the NF-κB pathway (Xu et al., 2022).

SARS-CoV-2 can also regulate NLRP3 through the second activation phase. Furthermore, ORF3a can use NEK7 and K^+^ efflux to trigger the NLRP3 inflammasome activation, resulting in the conversion of IL-1β precursors into mature IL-1β (Xu et al., 2022). Studies have revealed that the N proteins of SARS-CoV-2 interact with the inflammasome pathway to induce the transcription of NLRP3, pro-IL-1β and pro-IL-18 and to promote inflammasome assembly leading to active IL-18 and IL-1β release and accelerating inflammation (**Figure 2**) (Pan et al., 2021). Furthermore, some studies have revealed that the N protein of SARS-CoV-2 may activate NLRP3 by initiating the complement cascade pathway by forming the Mannan-binding lectin (MBL; alias: mannose-binding lectin) and MBL-associated serine protease 2 (MBL-MASP-2) complex. The complement cascade induced by N protein-MBL-MASP-2 may activate the NLRP3 inflammasome via the different functions of C3a, C5a, and membrane attack complex (MAC) (Asgari et al., 2013; Sluyter, 2017; Magro et al., 2020; Ratajczak and Kucia, 2020; Zhao et al., 2021). The insertion of MAC triggers Ca^2+^ influx and increases Ca^2+^ levels in the cytoplasm, thereby inducing mitochondrial damage and activating the NLRP3 inflammasome (Triantafilou et al., 2013a).

#### 2.1.2 Flavivirus

Flaviviruses remain a major public health concern worldwide. Dengue virus (DENV) and Zika virus (ZIKV) are two important pathogens in the genus *Flavivirus*, which comprise enveloped, positive, and approximately 11 kb single-stranded RNA (Xie et al., 2021). The encoded protein comprises three structural proteins: capsid (C), pre-membrane (prM), and envelope (E) in the N-terminal region and seven nonstructural proteins (i.e., NS1-NS2A-NS2B-NS3-NS4A-NS4B-NS5) in the C-terminal region (Chambers et al., 1990; Javed et al., 2018; Ngono and Shresta, 2018; Xie et al., 2021). The viral glycoprotein E binds to cell receptors and initiates endocytosis-mediated internalization. After the process of virus uncoating in the cytoplasm, the genomic RNA undergoes subsequent replication and transcription to produce new viral RNA and proteins. Finally, they are assembled into new virus particles and released (Mukhopadhyay et al., 2005; Barrows et al., 2018; Diosa-Toro et al., 2020; van Leur et al., 2021).

There are four primary dengue virus serotypes of DENV: DENV-1 to 4; they cause mild fever to severe dengue hemorrhagic fever or dengue shock syndrome, which is potentially life-threatening (Diamond and Pierson, 2015; Mustafa et al., 2015; Harapan et al., 2020). On the other hand, ZIKV infection can result in considerable neurological damage, including congenital Zika virus syndrome (Cao-Lormeau et al., 2016; Polonio and Peron, 2021).

##### 2.1.2.1 DENV virus infection and NLRP3 inflammasome

Dengue fever may occur because of a cytokine storm (de-Oliveira-Pinto et al., 2012). Patients with severe dengue have higher active IL-1β levels in their blood and gene expression profiles; this indicates its role in disease severity in clinical settings (Bozza et al., 2008; Pan et al., 2019b). Active IL-1β potentially recruits neutrophils to the inflammation site to help to clear DENV infection (Niu et al., 2019).

During host cell infection with DENV, first, signal 1 is activated: cytoplasmic pattern recognition receptors sense DENV viral RNA and trigger the NF-κB signaling pathway, thereby upregulating the transcription of pro-caspase-1, the NLRP3 inflammasome, pro-IL-1β, and pro-IL-18 (Shrivastava et al., 2020a). Many DENV viral proteins exert a strong effect on NLRP3 inflammasome activation and induce host immune responses. For example, in THP-1 cells (human monocyte leukemia cell line), the EDIII domain of E protein activates the NLRP3 inflammasome via the NF-κB pathway, resulting in the transcription of pro-IL-1β and TNF-α (Khan et al., 2019).

DENV can also regulate NLRP3 via the second activation phase. For example, EDIII can promote IL-1β secretion by inducing ROS generation and potassium efflux in the second step leading to the activation of the NLRP3 inflammasome and caspase-1-mediated maturation of pro-IL-1β (Khan et al., 2019). Further understanding of the role of EDIII in regulating inflammatory responses will help to understand the pathogenesis of DENV infections. Furthermore, the nonstructural proteins NS2A and NS2B of DENV can trigger the activation of the NLRP3 inflammasome via ROS and Ca^2+^ influx (Shrivastava et al., 2020b). The M protein of DENV is important in the innate immune response of the host. The interaction of M protein with NLRP3 suggests its crucial role in inflammasome activation, active IL-1β release, and subsequent pathogenic responses (Pan et al., 2019a). Therefore, DENV viral proteins are crucial in regulating the innate inflammatory responses of the host.

##### 2.1.2.2 ZIKV virus infection and NLRP3 inflammasome

A study has reported that the levels of proinflammatory cytokines such as IL-1β, interferon-γ, and IL-8 are increased in patients with ZIKV infection (Tappe et al., 2016). ZIKV infection can increase the transcription of proinflammatory cytokines such as IL-1β and IL-6 by activating the NF-κB signaling pathway (Gim et al., 2019).

**Figure 3:**
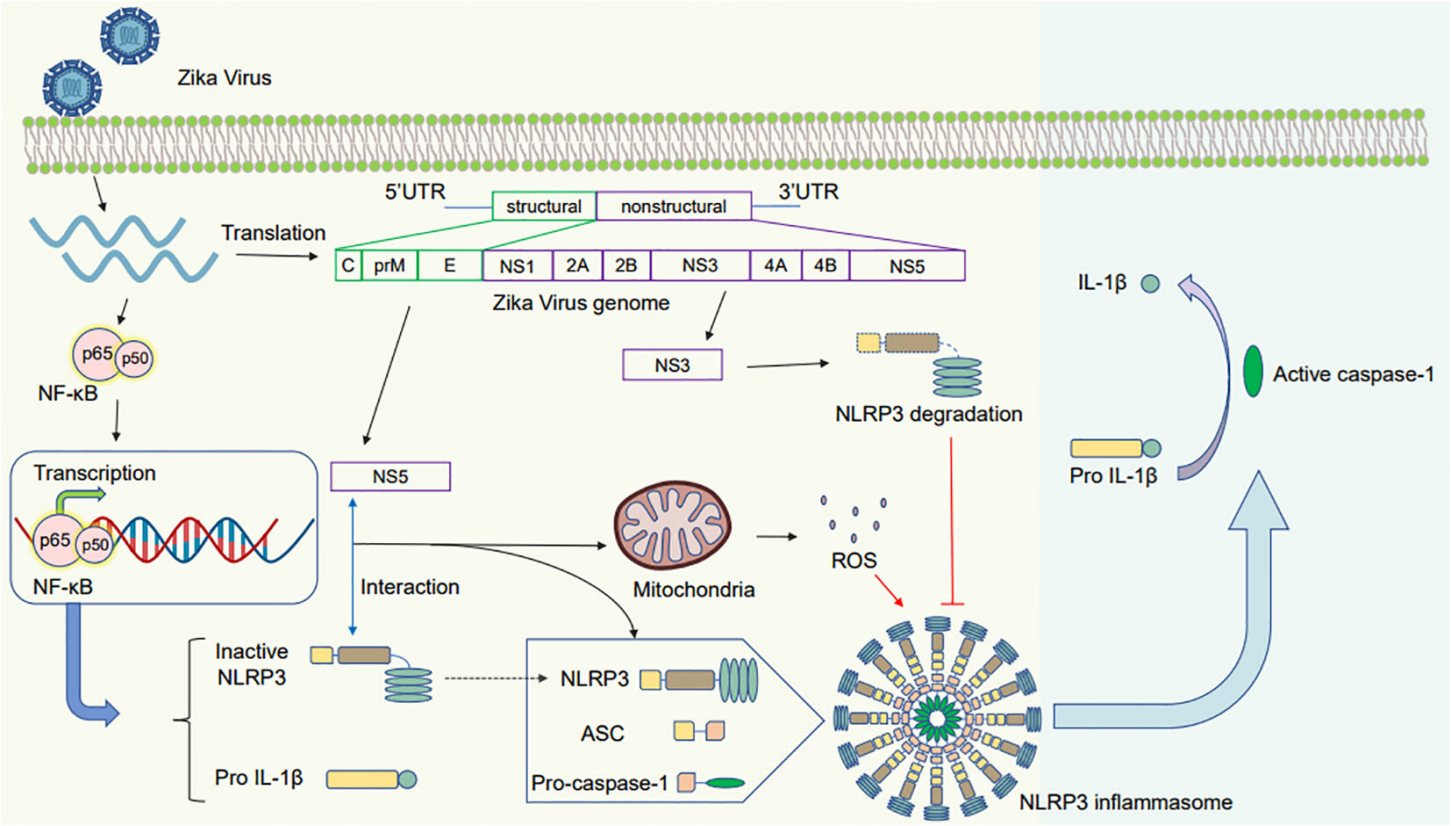
Schematic diagram of the mechanism by which Zika virus activates the NLRP3 inflammasome. After the Zika virus invades and infects the cells, it activates the NF-κB pathway, inducing gene expression to produce pro-IL-1β and NLRP3. (1) The NS5 protein interacts with NLRP3 to promote the assembly of the NLRP3 inflammasome. The induced mitochondrial ROS leads to pro-caspase-1 cleavage and IL-1β release (2): The NS3 protein inhibits the activation of the NLRP3 inflammasome by cleaving NLRP3, decreasing caspase-1 activation and mature IL-1β secretion.

Furthermore, ZIKV can regulate NLRP3 via the second activation phase. The NS5 protein plays a vital role in NLRP3 inflammasome activation (**Figure 3**). Wang et al. have suggested that the NS5 protein of ZIKV directly binds to the NLRP3 inflammasome for activation. NS5 promotes the assembly of the NLRP3–ASC inflammasome complex, resulting in pro-caspase-1 cleavage and secretion of active IL-1β (Wang et al., 2018). Furthermore, He et al. have discovered a novel role of the NS5 protein of ZIKV in regulating NLRP3 activation. NS5 induces ROS production and directly binds to NLRP3 to stimulate the assembly of the NLRP3 inflammasome complex triggering the maturation of pro-IL-1β (He et al., 2018; Wang et al., 2018). In addition, ZIKV results in oxidative stress in glial cells, resulting in NLRP3 inflammasome activation and mature IL-1β release for cell death (Tricarico et al., 2017).

In contrast, ZIKV NS3 protein can prevent the activation of NLRP3 inflammasome. ZIKV NS3 protein expression can decrease the extent of caspase-1 activation and active IL-1β secretion in macrophages, whereas its overexpression in 293T cells can lead to NLRP3 degradation (Gim et al., 2019).

#### 2.1.3 Enterovirus

Enterovirus 71 (EV71) belongs to the family *Picornaviridae* and is a positive-sense single-stranded RNA virus with a genomic length of 7.4 kb (Solomon et al., 2010; Wen et al., 2020). EV71 has four structural proteins, namely, VP1, VP2, VP3, and VP4, that are primarily involved in virus particle formation and virus binding to host cells. On the other hand, the nonstructural proteins 2A, 2B, 2C, 3A, 3B, 3C, and 3D are vital for virus infection, replication, and innate immune responses (Solomon et al., 2010; Wen et al., 2020). EV71 infection begins with receptor interaction. Then, it enters the cells via endocytosis and undergoes early uncoating. The viral RNA undergoes replication and transcription to generate new viral RNA and proteins. The resulting positive-sense viral RNA is loaded into the procapsid, eventually maturing into infectious virus particles (Solomon et al., 2010; Tan et al., 2014). EV71 has caused several epidemics worldwide. It can induce hand-foot-mouth disease, neonatal sepsis, meningoencephalitis, and pulmonary edema in children as well as other severe systemic disorders (McMinn, 2002; Ooi et al., 2010).

##### 2.1.3.1 EV71 virus infection and NLRP3 inflammasome

The infection of cells by EV71 can activate the NLRP3 inflammasome, which then causes the creation and release of proinflammatory cytokines including active IL-1β. This activation of the NLRP3 inflammasome may be involved in the antiviral defense mechanism of the host against EV71 infection (Wang et al., 2015).

EV71 can activate the NLRP3 inflammasome in the first step. EV71 uses cellular vimentin (VIM) to activate extracellular regulatory protein kinase (ERK) and translocate NF-κB. Ultimately, ERK phosphorylation triggers the activation of the NF-κB signaling pathway, which leads to the activation of the NLRP3 inflammasome and the transcription of IL-1β precursors. Therefore, Gong et al. concluded that EV71 activates the NLRP3 inflammasome via the VIM-ERK-NF-κB pathway (Gong et al., 2022).

EV71 can also regulate NLRP3 via a second activation phase. The nonstructural protein 3D of the virus plays a role in activating NLRP3 inflammasome-mediated transcription of pro-IL-1β. The 3D protein functions as an RNA-dependent RNA polymerase in EV71 and directly interact with the LRR and NACHT domains of NLRP3 to trigger the formation of the 3D–NLRP3–ASC inflammasome complex (Wang et al., 2017; You et al., 2020). Recent studies have reported that methylation of adenosine in RNA molecules generating N6-methyladenosine (m6A RNA) plays an important role in regulating the activation of the NLRP3 inflammasome during EV71 infection. The depletion of fat mass and obesity-associated protein can enhance the mRNA stability and expression of thioredoxin-interacting protein (TXNIP), thereby enhancing ROS production and NLRP3 inflammasome activation (Qayyum et al., 2021; Hu et al., 2023).

On the other hand, the EV71 viral proteins has a role in inhibition of NLRP3 activation. The nonstructural proteins 2A and 3C of EV71 inhibit NLRP3 inflammasome activation by lysing it (Wang et al., 2017). 2A and 3C can cleave NLRP3 at the G493–L494 and Q225–G226 junction, respectively, to prevent inflammasome activation. Furthermore, the 3C protein of EV71 interacts with NLRP3 and inhibits the secretion of active IL-1β in mammalian cells (Wang et al., 2015; Xiao et al., 2019). In summary, EV71 virus proteins exhibit dual roles in NLRP3 interaction.

#### 2.1.4 Retrovirus

Retroviruses comprise positive-sense single-stranded RNA genomes that infect cells via reverse transcription and genomic integration (Hu and Hughes, 2012; Zhang et al., 2018). The characteristic of retroviruses is that the virus-encoded reverse transcriptase reversely transcribes the viral RNA into DNA and integrates it into the chromosomes of host cells. Subsequently, all structural and regulatory proteins are transcribed and translated (Zhang et al., 2018). After viral protein and genome assembly, new virions bud out (Krebs et al., 2021). HIV, a well-known retrovirus, has considerably affected humanity since the discovery of acquired immunodeficiency syndrome in the early 1980s (Lucas and Nelson, 2015). HIV infection severely damages the immune system, and the virus targets critical CD4 T lymphocytes, resulting in severe cell damage and impairing immune function (Fanales-Belasio et al., 2010; Doitsh et al., 2014). HIV-1 and HIV-2 are two types of HIV. HIV-1 accounts for most infections. On the other hand, HIV-2, originating from West Africa, is less prevalent and less virulent than HIV-1 (Sharp and Hahn, 2011; Visseaux et al., 2019).

##### 2.1.4.1 HIV virus infection and NLRP3 inflammasome

HIV can interact with the NLRP3 inflammasome. Furthermore, HIV infection can trigger NLRP3 inflammasome activation, leading to the production and release of proinflammatory cytokines, including IL-1β (Reis et al., 2019; Zhang et al., 2019). A previous study has suggested that monocytes from HIV-1-infected individuals produce more IL-1β than those from noninfected individuals, indicating that HIV-1 triggers NLRP3 inflammasome activation (Jalbert et al., 2013).

HIV-1 can activate the NLRP3 inflammasome via the first activation step. HIV-1 activates the NLRP3 inflammasome in primary human monocyte-derived macrophages via the NF-κB pathway, promoting IL-1β secretion by increasing the amount of its precursor in the cell (Hernandez et al., 2014). Furthermore, HIV viral proteins can activate the NLRP3 inflammasome. For example, when it comes to HIV-1 replication and infection, viral protein R (Vpr) plays a crucial role as an accessory protein. Vpr can induce the activation of proinflammatory markers such as TNF-α and the NF-κB signaling pathway (Li et al., 2020). In addition, the HIV-1 transactivator of transcription (Tat) protein can prime the NLRP3 inflammasome by activating NF-κB. Subsequently, the interaction the interaction of the Tat protein with the inflammasome leads to caspase-1 maturation and IL-1β release (Demarchi et al., 1996; Chivero et al., 2017).

HIV can also regulate NLRP3 via a second activation phase. Tat can induce inflammasome activation by regulating Ca^2+^ flux (Benelli et al., 2000; Lee et al., 2012). HIV-1 RNA can be recognized by the pattern recognition receptor protein kinase RNA-activated (PKR), triggering the activation of the NLRP3 inflammasome by inducing ROS generation and activating the MAP kinases ERK1/2, JNK, and p38 (Stunnenberg et al., 2021). Furthermore, during the virus attachment step, the HIV-1 envelope glycoprotein causes extracellular ATP and K^+^ efflux by associating with PNX1, leading to NLRP3 activation (Séror et al., 2011; Paoletti et al., 2019; He et al., 2020).

### 2.2 Negative-sense single-stranded RNA virus

#### 2.2.1 Influenza virus

At present, influenza is an infectious disease worldwide and a serious global health concern; approximately 3 million to 5 million severe cases and 290,000–650,000 deaths are reported each year (Kim et al., 2022). Influenza viruses belong to the family *Orthomyxoviridae* and contain a negative-sense single-stranded RNA genome (Bouvier and Palese, 2008; Te Velthuis and Fodor, 2016). Human influenza viruses come in three different types: Influenza A, B, and C viruses (IAV, IBV, and ICV); they are the pathogens of the human respiratory disease influenza (Blut and Hemotherapy, 2009; Hutchinson, 2018). IAV and IBV have caused significant morbidity and mortality worldwide as well as an economic burden; in contrast, ICV causes a mild respiratory disease that rarely generates epidemics, mostly in children (Te Velthuis and Fodor, 2016). The virus binds to the cell surface receptors containing sialic acid and enters the cell via endocytosis. The viral core is released into the nucleus, where the viral genome is transcribed into mRNA and subsequently translated into viral proteins. Thereafter, the viral proteins and newly synthesized RNA assemble into new virus particles. After the maturation of the virus particles, they are released via membrane fusion or penetration (Watanabe et al., 2010; Te Velthuis and Fodor, 2016).

##### 2.2.1.1 Influenza virus infection and NLRP3 inflammasome

Influenza often triggers inflammatory responses in the immune system of humans (Kuriakose and Kanneganti, 2017). During influenza, host immune cells release cytokines such as IL-1β and IL-6 in response to virus invasion. Among these cytokines, IL-1β is a vital proinflammatory cytokine that regulates immune and inflammatory responses. Virus components may function as signals that activate the NLRP3 inflammasome, the maturation of pro-IL-1β into their active forms (Allen et al., 2009; Tate and Mansell, 2018).

Influenza can regulate NLRP3 via the first step. The viral nucleoprotein (NP) can stimulate neighboring cells via TLR2 and TLR4 activating the NF-κB pathway and thereby inducing the production of pro-IL-1β and IL-6; this subsequently leads to the production of trypsin which can increase the infectivity of influenza virus (Kim et al., 2022). For the priming associated with NLRP3 inflammasome activation, TLRs and retinoic acid-inducible gene I (RIG-I) are critical. Influenza viral RNA can indirectly promote inflammasome assembly and the release of inflammasome-dependent cytokines by interacting with known RNA sensors such as TLR-7 and RIG-I (Ichinohe et al., 2009; Kuriakose and Kanneganti, 2017). After influenza virus invasion, viral ribonucleoproteins are released into the nucleus for transcription and replication. SsRNA is recognized by TLR7 and triggers the expression of NF-κB, pro-IL-1β, pro-IL-18, and other pro-inflammatory cytokines (Niu and Meng, 2023). Studies have shown a lack of IL-1β release by infecting influenza virus into TLR7-deficient bone marrow-derived DCs (Ichinohe et al., 2010). In addition, when the viral genome reaches the cytoplasm, the 5α-triphosphate dsRNA of influenza A virus can activate RIG-I, which is involved in NF-κB activation and pro-IL-18 and pro-IL-1β transcription (Cui et al., 2008).

Influenza can also regulate NLRP3 via a second activation phase. The M2 protein of the influenza virus is a proton-selective ion channel that is important in NLRP3 inflammasome activation. The M2 protein can activate the NLRP3 inflammasome in macrophages and dendritic cells and plays a role in viral pathogenesis (Ichinohe et al., 2010). The M2 protein promotes inflammasome activation by locating the acidified Golgi apparatus, promoting proton outflow, causing ion imbalance, causing K efflux binding with Na efflux, and producing ROS, thereby activating the NLRP3 inflammasome. Furthermore, it triggers the inflammasome by modulating ion flux or stimulating mitochondrial DNA release into the cytoplasm (Ichinohe et al., 2010; Pang and Iwasaki, 2011; Moriyama et al., 2019). Moreover, a study has reported that NLRP3 can be recruited into dispersed Trans-Golgi-Network (TGN), where it oligomerizes, alters the conformation, and recruits ASC to activate the inflammasome (Pandey and Zhou, 2022). The PB1-F2 protein of IAV activates NLRP3 via various mechanisms. The PB1-F2 protein is translocated into the mitochondrial intima through Tom40 channel; its accumulation decreases the potential of the mitochondrial intima (Δφm), accelerates mitochondrial fragmentation, and activates the NLRP3 inflammasome (Yoshizumi et al., 2014). The aggregated form of the C-terminal region of PB1-F2 protein can activate NLRP3 via mitochondrial autophagy, promoting mitochondrial ROS production and mitochondrial DNA release (McAuley et al., 2013; Wang et al., 2021). Furthermore, IAV RNA activates the NLRP3 inflammasome. The activation of the NLRP3 inflammasome by influenza virus RNA depends on lysosomal maturation and ROS production (Allen et al., 2009).

However, the NLRP3 inflammasome’s activation can also be inhibited by influenza viruses. For example, IAVs can inhibit NLRP3 inflammasome activation via viral proteins. The NS1 protein of IAV interacts with NLRP3 to inhibit single-speck formation required for NLRP3 and ASC-induced inflammasome activation; this results in decreased secretion of active IL-1β (Moriyama et al., 2016). Chung et al. have reported that NS1 overexpression can significantly disrupt the transcription of proinflammatory cytokines by inhibiting the activation of NF-κB. Furthermore, inflammasome NLRP3 activation is inhibited in NS1-expressing THP-1 cells (Chung et al., 2015).

#### 2.2.2 Ebola virus

Ebola virus (EBOV) is one of the deadly zoonotic epidemic viruses that can cause fatal systemic disease (Marcinkiewicz et al., 2014; Malvy et al., 2019). The Ebola virus genome is composed of single negative strand RNA; the genome size is about 19kb; and it belongs to the *filoviridae* family (Baseler et al., 2017). The main characteristics of patients are high fever, fatigue, body aches, gastrointestinal symptoms, abnormal inflammatory response, immune suppression, large fluid and electrolyte loss, and high mortality (De Clercq, 2015; Malvy et al., 2019). When a virus infects, the virus first binds to the host cell membrane, triggering endocytosis of the virus particles. The particle envelope fuses with the endosomal membrane, thereby releasing the ribonucleoprotein complex into the cytoplasm. In the cytoplasm the viral genome is replicated and transcribed into mRNA. The viral proteins are then translated into the cytoplasm and into the endoplasmic reticulum. Mature daughter ribonucleoprotein complexes and viral proteins are transported to the plasma membrane. Finally, mature virions are released in the form of budding (Sakurai, 2015; Baseler et al., 2017; Jacob et al., 2020).

##### 2.2.2.1 Ebola virus infection and NLRP3 inflammasome

The immune response and inflammatory cascade of the innate immune system are key factors in the pathogenesis and mortality of EBOV (Leroy et al., 2001). The release of proinflammatory cytokines IL-1β, TNF-α, and IL-5 was found in asymptomatic individuals (Leroy et al., 2001). In addition, when EBOV infects monocytes, it also leads to the maturation and secretion of the pro-inflammatory cytokine IL-1β (Ströher et al., 2001).

It has been reported that Ebola virus-like particles stimulate the expression of type I interferon and proinflammatory cytokines through Toll-like receptors and interferon signaling pathways (Ayithan et al., 2014). Halfmann et al. found that EBOV stimulates the secretion of proinflammatory cytokines IL-1β and IL-18 by activating the NLRP3 inflammasome in a caspase 1-dependent manner (Halfmann et al., 2018). However, more research is still needed to study how EBOV activates the NLRP3 inflammasome to trigger the release of pro-inflammatory cytokines like IL-1β and the downstream role of IL-1β signal transduction.

#### 2.2.3 Respiratory syncytial virus

Respiratory syncytial virus (RSV) infection is a constant public health problem, and the impact on children, the elderly, and immunocompromised patients can be very significant (Yang et al., 2023). RSV is an enveloped, negatively-sense single-stranded RNA virus belonging to the *Paramyxoviridae* family (Collins et al., 2013). RSV has eight known structural proteins. Fusion protein (F), attached glycoprotein (G), small hydrophobic protein (SH), matrix protein (M), nucleocapsid protein (N), large protein (L), phosphoprotein (P), and M2 gene product M2-1, as well as two non-structural proteins (NS1 and NS1) (Borchers et al., 2013; Griffiths et al., 2017; Agac et al., 2023). The virus is mainly transmitted through close contact with saliva or mucus droplets. Common symptoms include fever, runny nose, cough, and chest tightness (Linder and Malani, 2017). RSV first binds to receptors on the surface of the host cell through endocytosis, or the viral F protein on its surface, and then the virus fuses with the cell membrane to enter the host cell. Viral RNA is released into the cytoplasm of the host cell. In host cells, viral RNA acts as a transcription template, producing mRNA. The mRNA is then translated into viral proteins. The newly synthesized viral protein and the replicating RNA are assembled into new viral particles, which bud out from the infected cell to complete the viral life cycle (Shang et al., 2021).

##### 2.2.3.1 Respiratory syncytial virus infection and NLRP3 inflammasome

Activation of the inflammasome pathway also plays an important role in RSV infection. Excessive inflammatory responses can lead to the release of pro-inflammatory cytokines, including IL-1β and IL-18, leading to lung inflammation and damage (Shen et al., 2018). Studies have shown that NLRP3, ASC, and caspase-1 are critical for IL-1β production during RSV infection (Segovia et al., 2012; Shen et al., 2018).

RSV can regulate NLRP3 via the first step. RSV activates NF-κB during infection (Sabbah et al., 2009). NF-κB signaling has been shown to be critical for NLRP3 expression during RSV infection (Shen et al., 2019). TLR2/MyD88/NF-κB signaling is required for pro-IL-1β and NLRP3 gene expression during RSV infection (Segovia et al., 2012). After the administration of NF-κB inhibitors in RSV-infected cells, the production of IL-1β decreased significantly (Segovia et al., 2012).

Respiratory syncytial virus can also regulate NLRP3 via a second activation phase. During RSV infection, activation of the NLRP3 inflammasome is dependent on K efflux and ROS production, followed by caspase-1-mediated maturation and secretion of IL-1β (Segovia et al., 2012; Yang et al., 2023). It has been shown that RSV SH viroporin induces membrane permeability to ions or small molecules that are essential for triggering the NLRP3 inflammasome. After RSV infection, RSV SH virus channel proteins accumulate in the Golgi within the lipid raft structure, possibly forming ion channels that trigger the translocation of NLRP3 from cytoplasm to Golgi apparatus. This leads to NLRP3 inflammasome activation as well as pro-IL1β transcriptional activation (Triantafilou et al., 2013b). It has also been reported that orosomucoid 1-like protein 3 (ORMDL3) can inhibit calcium pump function, resulting in increased calcium levels in the cytoplasm and decreased ER levels, thereby inducing ER stress (Cantero-Recasens et al., 2010). RSV may induce NLRP3 inflammasome expression by activating ORMDL3 overexpression (Cheng et al., 2023).

#### 2.2.4 Rift Valley Fever virus

Rift Valley Fever virus (RVFV) is a negative sense segmented single-stranded RNA virus with a size of about 12kb that belongs to the family *Phenuiviridae* and the genus *Phlebovirus* (Ahsan et al., 2016; Gaudreault et al., 2019). In humans, RVFV can cause a variety of disease manifestations, ranging from febrile illness to hemorrhagic fever and death (Ermler et al., 2014). The viral genome has three segments: the negative sense L (large) and negative sense M (medium) segments, and the double sense S (small) segment. These three segments encode multifunctional proteins. The S segment encodes nuclear protein (N) and non-structural protein S (NSs), the M segment encodes viral glycoprotein (Gn and Gc) and non-structural protein (NSm and a 78-kDa protein), and the L segment encodes viral RNA-dependent RNA polymerase (Baer et al., 2016; Wright et al., 2019; Tercero et al., 2021; Lean and Johnson, 2022; Ganaie et al., 2023). The virus first attaches to the host membrane and enters the host cell through endocytosis. The viral genome is released into the cytoplasm. Negative RNA is transcribed into positive mRNA encoding viral proteins, and positive RNA serves as a template for the synthesis of new negative RNA. The newly synthesized viral protein and the replicated genome are combined in the cytoplasm to form new viral particles, which are released when they mature (Wright et al., 2019).

##### 2.2.4.1 Rift Valley Fever virus infection and NLRP3 inflammasome

RVFV infection induces strong cytokines, which are essential for the recruitment of innate immune cells to the site of infection (Nair et al., 2023). RVFV infected-cells secrete IL-1β, which is involved in the NLRP3 inflammasome, ASC oligomerization, and caspase-1 maturation. It has been reported that RVFV activates the NLRP3 inflammasome by inducing the formation of an inflammasome complex containing NLRP3 and MAVS, where MAVS are localized to NLRP3 during RVFV infection, leading to the maturation and secretion of IL-1β (Ermler et al., 2014).

#### 2.2.5 Hantavirus

Hantavirus (HTNV) is a coated single-stranded negative sense RNA virus belonging to the *Bunyaviridae* family (Stock, 2008). The HTNV genome consists of three single-stranded negative sense RNA fragments: small (S), medium (M), and large (L) genome fragments encode four structural proteins (nuclear proteins N, glycoproteins Gn and Gc, and L proteins) (Muyangwa et al., 2015; Meier et al., 2021). HTNV can cause acute febrile illness in humans. Hemorrhagic Fever with Renal Syndrome (HFRS) caused in Asia and Europe, and Hantavirus Pulmonary Syndrome (HPS) caused in the Americas (Sargianou et al., 2012). Once Hantavirus enters the body, the virions bind to cell surface membrane receptors and enter the cell through endocytosis. The specific mechanism involves the transcription of the viral genome and the synthesis of viral RNA and viral proteins. The newly synthesized vRNA is coated with N protein to form ribonucleoprotein, which is then sent to the perinuclear membrane system. The synthesized viral proteins and genome are assembled into new viral particles inside the host cell. When the virion matures, it is released (Muyangwa et al., 2015; Meier et al., 2021).

##### 2.2.5.1 Hantavirus infection and NLRP3 inflammasome

HTNV infection causes cells to enter a stress condition and induces the production of inflammatory cytokines (Zhang et al., 2021). Studies have found that IL-1β is significantly elevated during HFRS. Induction of the human monocyte line THP-1 by HTNV revealed the secretion of IL-1β. The specific mechanism found that the induction of IL-1β by HTNV depended on the activation of caspase-1. Hantavirus thus induces the formation of the NLRP3 inflammasome in THP-1 cells, which may be an important factor in IL-1β levels in patients with HFRS (Ye et al., 2015).

### 2.3 Double-stranded RNA virus

#### 2.3.1 Reovirus

DsRNA viruses have complementary dsRNA. This virus family displays two distinctive features: (1) their virus genome typically comprises 10-12 double-stranded RNA segments, and (2) the virus contains a double capsid structure but lacks an envelope (Danthi et al., 2013). dsRNA viruses form a large group of RNA disease viruses, including reoviruses. Reoviruses comprise two concentric protein shells (the outer capsid and the core) that contain 10 segments of the dsRNA genome (Ahlquist, 2006; Abad and Danthi, 2020). The infectious life cycle of reovirus starts with the attachment of the viral protein σ1 to sialic acid residues on the target cell surface. Alternatively, proteolysis by extracellular proteases leads to the formation of infectious subvirion particles; these particles directly enter the cell via membrane penetration. Thereafter, transcriptionally active virus core particles are released into the cytoplasm. After virus replication and assembly, mature virus particles are released (Comins et al., 2008; Gong and Mita, 2014; Lemay, 2018; Abad and Danthi, 2020).

##### 2.3.1.1 Reovirus virus infection and NLRP3 inflammasome

Reoviruses use the host protein EphA2 to counteract the activation of the NLRP3 inflammasome (Zhang et al., 2020). Zhang et al. have reported that reovirus infection of airway epithelial cells increases EPHA2-dependent NLRP3 phosphorylation; this inhibits the activation of the inflammasome by inhibiting the recruitment of other inflammasome components. Upon virus infection, EphA2^−/−^ mice exhibited increased inflammatory infiltration, resulting in the secretion of active IL-1β and IL-18 (Zhang and Ricci, 2020; Zhang et al., 2020).

## 3 The clinical significance of NLRP3 inflammasome activation in the viral diseases

The inflammasome plays a crucial role in sensing viral infections and related pathologies. The main physiological function of inflammasomes is to initiate immune responses and help maintain tissue homeostasis and repair (Spel and Martinon, 2021). Infection with RNA viruses can induce the production of inflammatory factors that can lead to fatal diseases (Choudhury et al., 2021). Overstimulation of innate immunity is the direct cause of persistent morbidity and death from pathogenic virus infection (Deng et al., 2023). In some cases, it may also be associated with the development and exacerbation of diseases, such as chronic viral infections or autoimmune conditions (Choudhury et al., 2021; Bonaventura et al., 2022). Targeting inflammasome or inflammasome-dependent cytokines such as IL-1β can be used as a therapeutic strategy (Iannitti et al., 2016). Therefore, clinically targeting the activation of the inflammasome as a therapeutic approach to control viral infections requires further in-depth research. This will aid in the development of effective treatment strategies for managing viral infections.

## 4 Therapeutic strategies targeting the NLRP3 inflammasome for anti-inflammatory effects in virus infection

In virus infections, NLRP3-mediated inflammation and cytokine storms are associated with disease severity; therefore, determining ways to regulate the inflammasome during virus infection is vital for decreasing inflammation and disease severity. As a result, studying and developing small-molecule drugs targeting the regulation of the NLRP3 inflammasome are significant.

Cannabidiol exerts anti-inflammatory and immunomodulatory activities in the lungs and can inhibit the cytotoxicity and inflammation induced by SARS-CoV-2 spike proteins in Caco-2 cell lines via the peroxisome proliferator-activated receptor gamma-dependent TLR4/NLRP3/caspase-1 signaling pathway (Corpetti et al., 2021).

Statins also exert anti-inflammatory effects, and studies have discussed their potential beneficial effects in patients with COVID-19. Statins improve cytokine storms by inhibiting many molecular mechanisms, including the NF-κB pathway and NLRP3 inflammasome (Rodrigues-Diez et al., 2020).

Berberine is an isoquinoline alkaloid extracted from Chinese herbs. Liu et al. have reported that berberine inhibits the activation of the NLRP3 inflammasome induced by influenza virus in macrophages by inducing mitophagy and decreasing mitochondrial ROS (Liu et al., 2020b).

In addition, probenecid and AZ11645373 can target the P2X7 receptor signaling pathway and inhibit the responses of the NLRP3 inflammasome during IAV infection, subsequently limiting excessive inflammation and illness during influenza (Rosli et al., 2019).

ERK and NF-κB inhibitors also play vital roles in regulating the NLRP3 inflammasome during virus infection (Wei et al., 2016; Gong et al., 2022). The EV71-mediated NLRP3 inflammasome can be activated via the VIM–ERK–NF-κB pathway. As previously described, EV71 induces the activation of the NF-κB signaling pathway, which leads to the activation of the NLRP3 inflammasome and the transcription of IL-1β and IL-18 precursors. Therefore, VIM plays an important role in EV71-induced ERK phosphorylation, which triggers the activation of the NF-κB signaling pathway (Gong et al., 2022). PD098059 is a p-ERK inhibitor that significantly inhibits VIM-mediated ERK1/2 phosphorylation in EV71-infected cells, thereby preventing inflammasome activation (Gong et al., 2022). On the other hand, caffeic acid phenethyl ester is an NF-κB inhibitor that regulates the NLRP3 inflammasome after virus infection (Lee et al., 2016; Gong et al., 2022).

Disulfiram (DSF) plays an essential role in NLRP3 inflammasome-associated diseases (Deng et al., 2020; Huang et al., 2021). Deng et al. have reported that DSF can effectively inhibit NLRP3 inflammasome activation and active IL-1β release. Mechanistically, DSF prevents lysosomal rupture and subsequent cathepsin B release into the cytoplasm. Furthermore, it decreases mitochondrial-independent ROS production. Cathepsin B and ROS are important upstream signals for activating the NLRP3 inflammasome (Deng et al., 2020).

MCC950, a potent and selective NLRP3 inhibitor, is active in mice and human cells *in vitro*. It can specifically act on the NRLP3 inflammasome and does not inhibit the NLRP1, AIM2, or NLRC4 inflammasome. MCC950 targets the NLRP3 inflammasome by blocking caspase-1-dependent IL-1β processing by inhibiting NLRP3-induced ASC oligomerization. Therefore, future clinical development of MCC950 or its derivatives may lead to new anti-inflammatory therapies (Coll et al., 2015). For example, MCC950 has been used to inhibit NLRP3 inflammasome activity after RSV infection (Malinczak et al., 2021).

CY-09 specifically inhibits the activation of the NLRP3 inflammasome. CY-09 directly binds to the ATP-binding motif of the NACHT domain of NLRP3 and inhibits its ATPase activity, thereby inhibiting the assembly and activation of the NLRP3 inflammasome. Therefore, CY-09 provides a direct and selective small-molecule NLRP3 inhibitor for the targeted treatment of NLRP3-associated diseases (Jiang et al., 2017).

In summary, we can use various approaches to inhibit the activation of the NLRP3 inflammasome during virus infections. For example, the NLRP3 inflammasome can be inhibited by inhibiting upstream signaling molecules, inflammasome assembly, caspase-1 activation, GSDMD cleavage, etc. Furthermore, the use of inhibitors targeting the P2X7 receptor, K^+^ efflux, ROS, or ATPase activity of NRLP3 is an efficient approach (Lamkanfi et al., 2009; Hu et al., 2014; Jiang et al., 2017). All the abovementioned methods may contribute to antiviral immune defense strategies that inhibit inflammasome activation in virus-infected hosts.

## 5 Conclusions

Virus infections threaten public health and economic growth worldwide. During virus invasion, the excessive activation of the NLRP3 inflammasome can lead to a cytokine storm that increases virus infection and damages the tissues. However, insufficient activation of the NLRP3 inflammasome prevents the organism from responding to the virus invasion, facilitating the survival of harmful microorganisms and resulting in infections and diseases (Abdin et al., 2020; Sharma and Kanneganti, 2021). Therefore, the NLRP3 inflammasome is a double-edged sword in host defense against virus infection.

The regulation of the NLRP3 inflammasome plays a crucial role in controlling inflammation. Summarizing studies on NLRP3 inflammasome activation during RNA virus infections, understanding virus-induced pyroptosis, and exploring approaches to inhibit virus-triggered activation can help to understand the pathogenesis of inflammatory diseases caused by RNA virus infections and discover new therapeutic targets. This will, in turn, help control virus infection and develop therapies based on the severity of the illness.

## Author contributions

ZY: Writing – original draft, Writing – review & editing. XZ: Writing – original draft, Software. YG: Writing – original draft. YL: Writing – original draft. L-ML: Writing – original draft. YLL: Writing – original draft. YKL: Writing – original draft, Supervision. GY: Supervision, Writing – original draft, Writing – review & editing. PW: Supervision, Writing – original draft, Writing – review & editing. XC: Conceptualization, Funding acquisition, Supervision, Validation, Writing – original draft, Writing – review & editing.
